# The Interplay Between Picky Eating, Other Eating Behaviors, and Obesity Indicators in Preschool Children

**DOI:** 10.1002/fsn3.70967

**Published:** 2025-09-24

**Authors:** Melika Mahmoudizadeh, Mina Babashahi, Milad Rajabzadeh‐Dehkordi, Mehran Nouri, Shiva Faghih

**Affiliations:** ^1^ Department of Community Nutrition, School of Nutrition and Food Sciences Shiraz University of Medical Sciences Shiraz Iran; ^2^ Nutrition Research Center Shiraz University of Medical Sciences Shiraz Iran; ^3^ Student Research Committee Shiraz University of Medical Sciences Shiraz Iran; ^4^ Infertility and Reproductive Health Research Center, Health Research Institute Babol University of Medical Sciences Babol Iran

**Keywords:** food avoidance, obesity, picky eating, preschool children

## Abstract

Picky eating (PE) during early childhood strongly affects children's food preferences. This research aimed to assess the association of eating behaviors with obesity indices among preschoolers. Our cross‐sectional analysis included 436 pre‐school children. For data collection, parents were interviewed face‐to‐face, using the Child Eating Behavior Questionnaire and the Food Frequency Questionnaire for children eating behaviors and dietary intakes, respectively. Additionally, their BMI‐for‐age *z*‐score, waist circumference, and waist‐to‐height ratio were measured. The association between eating behaviors and obesity indicators was evaluated via logistic regression models. The prevalence of overweight and obesity (OW/OB) was 12.4%. Higher intake of ultra‐processed foods and lower intakes of energy and staple meals were found among children with high PE. PE was associated with 76%, 67%, and 60% lower risk of OW/OB, abdominal, and central obesity, respectively (OR = 0.238, 95% CI 0.135–0.420 OW/OB; OR = 0.332, 95% CI 0.220–0.502 abdominal obesity; OR = 0.398, 95% CI 0.278–0.571 central obesity). All food avoidance behaviors were negatively associated with the three obesity indicators. In contrast, food approach behaviors, except for desire to drink, had positive associations with obesity risk. All three obesity indicators were negatively associated with PE and food avoidance behaviors but positively associated with food approach behaviors. Since picky eaters commonly have unhealthy diets, being attentive to children's emotional and sensory challenges during mealtime could help nurture a healthier relationship with food.

## Graphical abstract

This study investigated the relationship between picky eating, other eating behaviors, and obesity indicators in preschool children. Picky eating and food avoidance behaviors were negatively associated with overweight, abdominal, and central obesity, while food approach behaviors increased obesity risk. Despite picky eaters’ tendency toward unhealthy diets, addressing emotional and sensory challenges during mealtime may foster healthier eating patterns and prevent obesity.

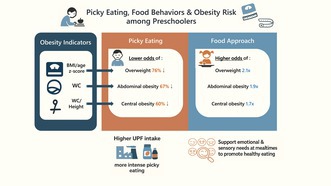

## 1 Introduction

Preschool period is an important time in creating children's eating habits and dietary patterns (Van den Brand et al. 2023). Child's eating behaviors, such as picky eating, can be influenced by both genetics and environmental factors (Nas et al. 2025). Picky eating is a common dietary behavior in young children which may cause disruption in healthy eating behaviors (Cole et al. 2017). This behavior can increase parents' stress and has negative effects on families (Goh and Jacob 2012). Picky eating is child's refusal to eat familiar or unfamiliar foods, reluctance to try new foods, and strong desire for certain foods, negatively affecting both parents' and children's behaviors (Taylor et al. 2015). It can also result in limited food variety and inadequate food intake (Taylor et al. 2016). Due to the differences in the definition of picky eating and the population of the studies, the prevalence of this behavior has been reported in a wide range of 5%–47% (Pernilla Sandvik et al. 2019; Tharner et al. 2014).

Children may not receive enough healthy and nutritious foods for their common growth due to some reasons, including lack of information about these foods, cultural reasons in families, and limited access to nutritious foods (Black et al. 2020; Scaglioni et al. 2018). Picky eating often begins when a child is 2 years old. Evidence regarding the peak age of picky eating is different. Some studies have stated the peak age of this behavior at 3 years old, but one study has shown that the peak of picky eating occurred at the age of 6 (Taylor et al. 2015; Van Tine et al. 2017). Food habits and preferences developed during childhood often continue in adolescence and can even persist into adulthood (Pesch et al. 2020). Children with continuous picky eating may suffer from developmental disorders in adolescence and even eating disorders during adulthood (Cardona Cano et al. 2016). This indicates the long‐term effect of eating behaviors on individuals' dietary habits and body weight, which emphasizes the need for early interventions (Dubois et al. 2022).

Picky eating can limit the variety of children's diet, leading to inadequate intake of nutrients and a reduction in the range of food choices (da Costa et al. 2022; Taylor and Emmett 2019). Some studies have shown that picky eaters consume less fruit and vegetables but consume more fat and sweets than normal children (Brown et al. 2018; Volger et al. 2013). As these behaviors continue, children are at risk of nutritional deficiencies and may become underweight (Antoniou et al. 2016), which can lead to reduced child growth and increased risk of diseases (Ibrahim et al. 2017). Overall, these behaviors can cause stress and anxiety in families and also have negative effects on family relationships (Goh and Jacob 2012).

Considering the importance of evaluating eating behaviors, the high prevalence of picky eating in young children, and the presence of limited studies about the relationship between these eating behaviors and weight status and dietary intakes of Iranian children, the current study aimed to investigate the association of picky eating and other eating behaviors with weight indices among Iranian preschoolers.

## 2 Methods and Materials

### 2.1 Study Design

The current cross‐sectional study was performed in 16 health care centers from 11 municipalities and 4 kindergartens from 4 educational districts, from July to December 2023 in Shiraz, Iran. Children were selected from each of these locations using the stratified sampling method. The number of 439 children with an age range of 2–6 years participated in this study (330 children from health care centers and 109 children from kindergartens were recruited). Questionnaires were filled out by parents through face‐to‐face interviews. Some criteria such as having a sibling relationship with the main participant (*n* = 4), incomplete information (*n* = 3), being non‐Iranian (*n* = 2), or suffering from metabolic diseases (*n* = 1) were considered exclusion criteria.

Based on a previous similar study and the power of 0.9 and first type error = 0.05, the minimum sample size was estimated as 265 children. In this study, we selected more children to increase the accuracy.

After explaining the purpose of the study, parents completed written informed consents. The ethics committee of Shiraz University of Medical Sciences approved the protocol of the study (IR.SUMS.SCHEANUT.REC.1401.137).

### 2.2 Data Collection

Demographic information of the children (such as sex, age, physical activity, birth weight, screen time, duration of breastfeeding, and attendance to kindergarten) and parents (including age, education, job, and income) were collected. Parents' heights and weights were self‐reported.

Anthropometric measurements of the children were recorded by two nutritionists. Weight was measured by a digital scale (Mi Body Composition Scale 2) with an accuracy of 0.01 g. A non‐stretchable tape attached to a wall was used to measure the children's height. Also, waist circumference (WC) (midpoint of the last rib and the superior iliac crest) was measured using a non‐stretchable tape to the nearest 0.1 cm.

In order to assess the weight status of the children, the *z*‐score of BMI for age (BMI/A) was used (Organization 2006), which was calculated by WHO Anthro (version 3.2.2.1, 2023) or WHO Anthro plus (version 1.0, 2014). For children with the age of 2–5 years old, BMI/A *z*‐score was categorized as: *z* < −2: wasted; −2 ≤ *z* ≤ 2: normal weight; 2 < *z* ≤ 3: overweight; z > 3: obese. The weight status classification for children between 5 and 6 years was as follows: *z* < −2: wasted; −2 ≤ z ≤ 1: normal weight; 1 < *z* ≤ 2: overweight; *z* > 2: obese (De Onis and Lobstein 2010). WC more than the 75th percentile was considered abdominal obesity (Fernández et al. 2004). Central obesity was determined as waist‐to‐height ratio (WHtR) more than 0.5 (Ejtahed et al. 2019).

Level of physical activity of the children was evaluated by “Physical Activity Questionnaire for Children (PAQ‐C)”. This questionnaire contained 9 items, and each item had 5‐point scale. The lowest and highest physical activity scores for each item were between the range of 1 (lowest) to 5 (highest) points. The mean of these items was the total PAQ‐C score (Zameni et al. 2020).

Picky eating and other eating behaviors were investigated using the Children's Eating Behavior Questionnaire (CEBQ), established by Jane Wardle and colleagues (Wardle et al. 2001). The Iranian version of the CEBQ demonstrated good internal consistency (83%), test–retest reliability (86%), and construct validity (seven extracted factors accounted for 62.8% of the total variance) in children's eating styles (Dasht Bozorgi and Askary 2017). CEBQ comprises 35 items with a 5‐point Likert scoring (ranging from 1 = “never” to 5 = “always”). Parents were asked to select the most appropriate response (“never,” “rarely,” “sometimes,” “often,” or “always”) for each item. Seven items were reverse scored. This multidimensional questionnaire originally assesses eight behaviors: (1) enjoyment of food, (2) emotional overeating, (3) food responsiveness, (4) desire to drink, (5) food fussiness, (6) satiety responsiveness, (7) slowness in eating, and (8) emotional undereating. The first four behaviors are “food approach” subscales, indicating a child's enthusiasm for food. The remaining four behaviors reflect a child's dislike of food, considered as “food avoidance” subscales. To measure selective eating more comprehensively, a composite picky eating score was created by summing scores of food fussiness, slowness in eating, and satiety responsiveness, plus reversed scores of food responsiveness and enjoyment of food subscales (Taylor et al. 2015).

### 2.3 Dietary Assessment

Children's dietary intakes were evaluated by a validated food frequency questionnaire (FFQ) (with 147 items) (Mirmiran et al. 2010) filled by parents. Frequency and quantity of each food item were recorded; then gram values were assigned to each. To find intakes of macronutrients, micronutrients, and daily energy intake, NUTRITIONIST IV software (version 7.0; N‐Squared Computing, Salem, OR, USA) was utilized.

The Healthy Eating Index (HEI) was used for diet quality assessment. It included adequacy components (direct score) and moderation components (reverse score). Adequacy components were greens and beans, total vegetables, total fruit, whole fruit, fatty acids, total proteins, plant proteins, dairy, and whole grains. Moderate components were refined grains, saturated fats, sodium, and added sugar. The total score of this index was between 0 and 100. All groups received a score of 0–5, except fatty acids and moderation components, which got a score of 0–10. The higher scores indicate a greater consumption of healthier foods based on the Dietary Guidelines for Americans (DGA) (Shams‐White et al. 2023).

Based on the NOVA classification, foods undertaken high processing levels have been known as Ultra‐processed foods (UPFs) since 2010 (Monteiro et al. 2010). UPFs are featured by excessive amounts of energy, sugar, unhealthy fats, and industrial additives, exerting harmful effects on individuals' health. For the calculation of UPFs, FFQ items were categorized in one of the four groups of NOVA classification: (1) unprocessed or minimally processed foods, (2) processed culinary ingredients, (3) processed foods, and (4) UPFs. Then, the whole energy content of UPFs items was divided by daily energy intake and multiplied by 100 to calculate the energy contribution of UPFs.

### 2.4 Statistical Analysis

All analyses were done by SPSS software version 25.0 (SPSS Inc., Chicago IL, USA) and visualized with GraphPad Prism 10.4 (Boston, MA, USA). Normal distribution of the variables was assessed by the Kolmogorov–Smirnov test. For normally distributed variables, mean and standard deviation (SD), and for other variables median and interquartile range (IQR) were used. To compare qualitative variables, chi‐squared test was applied. Independent samples *t*‐test was done to compare the mean value of two groups, and Mann–Whitney *U* test was utilized to compare non‐parametric variables. The scores of picky eating across UPFs tertiles were analyzed using Kruskal‐Wallis' test. The association between picky eating behaviors and obesity was evaluated by logistic regression model, which was adjusted for age, birth weight, breastfeeding duration, kindergarten attendance, family income, mother's BMI, and HEI (Table S1). A *p*‐value < 0.05 indicated a level of statistical significance.

## 3 Result

### 3.1 General Information

Our participants consisted of 436 children with a median age of 5 (2) years, including 52.1% girls (*n* = 227). 31.9% of the participants were the only children and nearly half (50.2%) belonged to families within the lowest income bracket (Table 1).

**TABLE 1:** Features of study's population based on low and high picky eating.

Characteristics	Total (*n* = 436) (1.32–4.93)	Picky eating		*p* value
		Low (*n* = 217) (≤ 3.17)	High (*n* = 219) (> 3.17)	
Age (year)^a^	5 (2)	4.5 (2)	5 (2)	0.576
Sex, girl^b^	227 (52.1%)	108 (49.8%)	119 (54.3%)	0.340
Physical activity (score)^c^	2.80 ± 0.63	2.82 ± 0.61	2.78 ± 0.65	0.566
Weight (kg)^a^	17.1 (5.28)	17.52 (6.29)	16.7 (4.9)	**0.008**
Weight for age *z*‐score^a^	−0.27 (1.41)	0.07 (1.67)	−0.05 (1.31)	< **0.001**
Height (cm)^a^	108 (15)	108.25 (17.5)	107 (13)	0.443
Height for age *z*‐score^c^	−0.10 ± 1.04	−0.00 ± 1.04	−0.21 ± 1.04	**0.033**
WC (cm)^a^	51 (6.5)	52 (7.38)	50 (5)	< **0.001**
WHtR^a^	0.48 (0.06)	0.49 (0.06)	0.47 (0.05)	< **0.001**
Birth weight^b^				
LBW (< 2500 g)	42 (9.6%)	18 (8.3%)	24 (11%)	0.284
NBW (2500–4000 g)	379 (86.9%)	189 (87.1%)	190 (86.8%)	
HBW (> 4000 g)	15 (3.4%)	10 (4.6%)	5 (2.3%)	
Screen time (min)^a^	240 (180)	210 (210)	240 (210)	0.094
Eating in front of a digital screen^b^				
Yes	311 (72.8%)	138 (65.4%)	173 (79.7%)	**0.001**
No	117 (27.2%)	73 (34.6%)	44 (20.3%)	
Kindergarten attendance^b^				
< 6 weeks	219 (50.2%)	119 (54.8%)	100 (45.7%)	0.055
6 weeks≤	217 (49.8%)	98 (45.2%)	119 (54.3%)	
Breastfeeding duration (month)^a^	20 (18)	20 (16.25)	14.56 (20)	**0.010**
Mother's age (year)^a^	36 (7)	35.5 (7)	36 (8)	0.852
Mother's BMI (kg/m^2^)^b^				
Underweight	3 (0.7%)	3 (1.4%)	0 (0%)	0.271
Normal weight	174 (40.0%)	83 (38.4%)	91 (41.6%)	
Overweight	163 (37.5%)	85 (39.4%)	78 (35.6%)	
Obese	95 (21.8%)	45 (20.8%)	50 (22.8%)	
Mother's education^b^				
Under diploma	53 (12.2%)	30 (13.9%)	23 (10.6%)	0.275
Diploma	144 (33.2%)	76 (35.2%)	68 (31.2%)	
Upper diploma	237 (54.6%)	110 (50.9%)	127 (58.3%)	
Mother's job^b^				
Household	341 (78.4%)	173 (80.1%)	168 (76.7%)	0.392
Employed	94 (21.6%)	43 (19.9%)	51 (23.3%)	
Family income per month (rial)^b^				
< 100 million	219 (50.2%)	114 (52.5%)	105 (47.9%)	**0.048**
100–200 million	160 (36.7%)	72 (33.2%)	88 (40.2%)	
200–300 million	25 (5.7%)	18 (8.3%)	7 (3.2%)	
≥ 300 million	32 (7.3%)	13 (6%)	19 (8.7%)	

*Note:* Significant values (*p* < 0.05) are in bold type.

Abbreviations: BMI, body mass index; HBW, high birthweight; LBW, low birthweight; NBW, normal birthweight; WC, waist circumference.

^a^ Using Mann–Whitney *U* test and values are median (IQR).

^b^ Using chi‐square tests for categorical variables and values are *n* (%).

^c^ Using independent sample t‐test and values are mean ± SD.

Children exhibiting high picky eating have been breastfed for a shorter duration (14.56 vs. 20 months; *p* = 0.01). The rate of eating in front of a digital screen was notably higher among these children (79.7% vs. 65.4%; *p* = 0.001). Coming from an underprivileged familyit was shown to be protective against picky eating, compared to a wealthier one (7.8% vs. 6% were from a rich family, 47.9% vs. 52.2% were from an underprivileged family; *p* = 0.048). No differences were found between the low and high picky eating groups in terms of maternal characteristics (Table 1).

**FIGURE 1:**
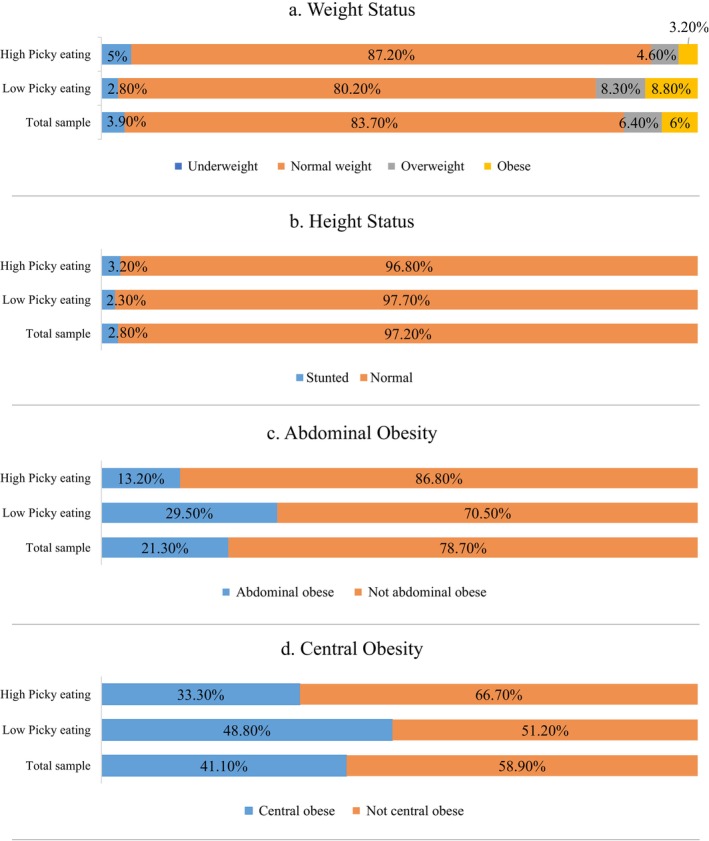
Relative frequencies for the categories of (a) BMI‐for‐age *z*‐score (weight status), (b) height‐for‐age (stunting), (c) waist circumference (abdominal obesity), and (d) waist‐to‐height ratio (central obesity) in the study's population based on picky eating degree.

The proportion of children with abnormal growth was identified as 3.9% underweight, 6.4% overweight, 6% obese, and 2.8% stunted. In total, 21.3% of the children had abdominal obesity and 41.1% had central obesity. Underweight and stunting were more prevalent, while overweight and obesity (OW/OB), abdominal, and central obesity were less prevalent among the high picky eating group compared to the low picky eating group (Figure 1).

Children identified as high picky eaters demonstrated more severe picky eating and food avoidance compared to low picky eaters (picky eating: 3.65 vs. 2.69; *p* < 0.001, food avoidance: 3.49 vs. 2.66; *p* < 0.001). All food avoidance behaviors including food fussiness, emotional undereating, satiety responsiveness, and slowness in eating were more common in the high picky eating group (food fussiness: 3.16 vs. 2.16; *p* < 0.001, emotional undereating: 3 vs. 2.75; *p* < 0.001, satiety responsiveness: 4 vs. 3; *p* < 0.001, slowness in eating: 4 vs. 2.75; *p* < 0.001). Conversely, they had lower scores of food approach and its subgroup behaviors including enjoyment of food, emotional overeating, and food responsiveness, in comparison to the low picky eating group (food approach: 2.09 vs. 2.75; *p* < 0.001, enjoyment of food: 2.75 vs. 4.25, *p* < 0.001, emotional overeating: 1 vs. 1.5; *p* < 0.001, food responsiveness: 1.6 vs. 2.4; *p* < 0.001). No differences were observed for desire to drink between the high and low picky eating groups (Table S2).

### 3.2 Dietary Intakes and Quality

Based on HEI score, lower diet quality was observed among high picky eaters compared to low picky eaters (54 vs. 55; *p* = 0.022). Their dietary content of energy (1873.7 vs. 2193.01 kcal; *p* < 0.001), carbohydrate (313.15 vs. 326.64 g; *p* < 0.001), fat (67.36 vs. 74.20 g; *p* < 0.001), SFA (22.19 vs. 24.36 g; *p* < 0.001), MUFA (21.55 vs. 24.09 g; *p* < 0.001), and fiber (26.6 vs. 33.34 g; *p* = 0.001), in addition to their consumption of water (828.1 vs. 946.4 mL; *p* = 0.025) and certain healthy food groups including refined grains (188.83 vs. 245.5 g; *p* = 0.020) and vegetables (149.1 vs. 217.43 g; *p* = 0.010), was lower. However, children in the high picky eating group consumed greater amounts of fast foods (12.42 vs. 10.62 g; *p* = 0.037), snacks (41.98 vs. 41.38 g; *p* = 0.001), and sugar‐sweetened beverages (71.42 vs. 64.52 g; *p* < 0.001). Also, their dietary intake of PUFA was significantly higher than that of the low picky eating group (14.63 vs. 14.13 g; *p* = 0.044) (Table 2).

**TABLE 2:** Dietary intakes of study's population based on low and high picky eating.

	Total (*n* = 436) (1.32–4.93)	Picky eating		*p* value
		Low (*n* = 217) (≤ 3.17)	High (*n* = 219) (> 3.17)	
HEI (score)	54 (50–60)	55 (50–60)	54 (49–59)	**0.022**
Dietary intakes				
Energy (kcal/days)	2040.67 (1605.39–2568.05)	2193.01 (1728.56–2806.15)	1873.70 (1504.20–2342.59)	< **0.001**
Protein (g/days)	65.83 (52.38–84.34)	73.17 (58.38–92.17)	60.16 (47.34–75.95)	0.066
Carbohydrate (g/days)	319.51 (300.10–338.85)	326.64 (301.39–344.72)	313.15 (296.61–329.64)	< **0.001**
Fat (g/days)	70.04 (54.03–89.82)	74.20 (55.57–95.25)	67.36 (52.07–85.87)	< **0.001**
SFA (mg/days)	23.45 (17.91–29.95)	24.36 (18.92–30.47)	22.19 (17.54–28.96)	< **0.001**
MUFA (mg/days)	22.38 (17.54–29.15)	24.09 (18.19–31.32)	21.55 (17.35–27.61)	< **0.001**
PUFA (mg/days)	14.44 (12.57–16.75)	14.13 (12.20–16.49)	14.63 (13.15–16.94)	**0.044**
Sodium (mg/days)	2352.08 (1830.32–2966.45)	2474.28 (1940.27–3123.58)	2223.73 (1706.62–2821.69)	0.392
Fiber (g/days)	29.99 (22.46–39.20)	33.34 (26.23–44.15)	26.60 (17.94–35.03)	**0.001**
Sugar (mg/days)	118.37 (89.46–153.36)	128.60 (100.11–172.16)	105.90 (78.20–136.70)	0.378
Water (mL/days)	946.40 (709.80–1183.0)	946.40 (709.80–1301.30)	828.10 (591.50–1183.0)	**0.025**
Food groups consumption				
Bread, rice, and pasta (g/days)	189.39 (138.00–274.63)	220.5 (161.51–301.84)	164.14 (116.28–234.32)	**0.006**
Whole grain (g/days)	14.13 (4.50–33.85)	17.88 (5.91–40.66)	11.28 (3.25–30.33)	0.482
Refined grain (g/days)	207.26 (153.13–291.10)	245.50 (167.37–331.19)	188.83 (144.90–250.28)	**0.020**
Low fat dairy (g/days)	105.61 (33.99–222.74)	110.85 (37.14–230.75)	98.57 (25.83–201.42)	0.971
High fat dairy (g/days)	196.74 (121.84–332.24)	2196.45 (106.54–304.99)	197.02 (131.04–350.81)	0.066
Meat (g/days)	50.19 (31.71–74.99)	56.45 (38.91–84.37)	43.24 (26.95–66.02)	0.103
Fish (g/days)	6.0 (0.98–13.49)	6.74 (1.43–14.45)	3.86 (0.73–12.74)	0.667
Egg (g/days)	26.7 (15.25–45.77)	26.7 (22.88–53.40)	22.88 (15.25–45.77)	0.502
Fruit (g/days)	735.53 (480.31–1058.26)	832.65 (577.86–1175.74)	649.90 (387.85–974.96)	0.161
Vegetable (g/days)	186.79 (115.13–288.54)	217.43 (144.71–324.94)	149.10 (92.82–259.63)	**0.010**
Legume (g/days)	34.26 (20.63–53.22)	39.54 (24.91–58.84)	32.15 (17.57–46.76)	0.135
Nuts (g/days)	9.5 (4.57–16.86)	10.95 (5.56–20.63)	8.04 (3.42–13.94)	0.935
Fast foods (g/days)	11.59 (5.70–18.70)	10.62 (4.35–18.64)	12.42 (8.02–18.81)	**0.037**
Snacks (g/days)	41.52 (23.8–70.0)	41.38 (23.46–69.69)	41.98 (24.10–70.47)	**0.001**
SSB (g/days)	67.75 (28.57–157.95)	64.52 (23.33–158.34)	71.42 (33.33–158.09)	< **0.001**
Oils (g/days)	10.15 (6.0–15.0)	12.0 (6.0–16.45)	8.89 (6.0–13.87)	0.075

*Note:* Data are presented as median (25th–75th). All *p* values were obtained from Mann–Whitney *U* test (adjusted for energy). Significant values (*p* < 0.05) are in bold type.

Abbreviations: HEI, healthy eating index; MUFA, monounsaturated fatty acids; PUFA, polyunsaturated fatty acids; SFA, saturated fatty acids; SSB, sugar‐sweetened beverages.

**FIGURE 2:**
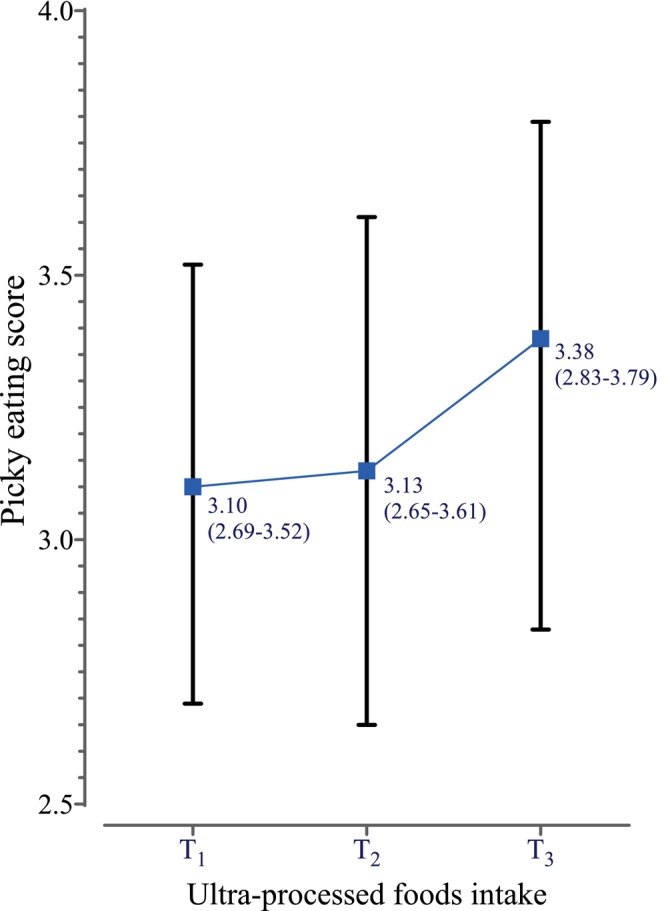
Picky eating scores across tertiles of ultra‐processed foods consumption. Children with the highest consumption of UPFs presented higher degrees of picky eating (*p*‐value = 0.032). Kruskal‐Wallis test was used to analyze the scores of picky eating across tertile of UPF consumption. The median (25th–75th) picky eating scores of tertiles are presented.

Children in the highest tertile of UPF consumption presented a higher median score of picky eating, compared to children in the lowest tertile (median (25th–75th) = 3.38 (2.83–3.79) T3 vs. 3.1 (2.69–3.52) T1; *p*‐value = 0.032) (Figure 2).

### 3.3 Association Between Eating Behaviors and Obesity Indices

Picky eating, food avoidance, emotional undereating, satiety responsiveness, and slowness in eating were negatively associated with all three obesity indicators: OW/OB, abdominal, and central obesity. Each one‐point increase in picky eating was associated with 76%, 67%, and 60% lower odds of OW/OB, abdominal, and central obesity, respectively (OW/OB: OR = 0.238; 95% CI 0.135–0.420, abdominal obesity: OR = 0.332; 95% CI 0.220–0.502, central obesity: OR = 0.398; 95% CI 0.278–0.571). Food fussiness was not associated with OW/OB, abdominal, and central obesity in univariate analysis (OW/OB: OR = 0.754; 95% CI 0.525–1.083, abdominal obesity: OR = 0.770; 95% CI 0.577–1.028). However, after adjusting for confounders, both associations became significant (v: OR = 0.590; 95% CI 0.387–0.899, abdominal obesity: OR = 0.673; 95% CI 0.491–0.922). Additionally, food fussiness was significantly associated with central obesity both before and after adjustment (univariate: OR = 0.565; 95% CI 0.439–0.727, multivariate: OR = 0.522; 95% CI 0.387–0.703) (Table 3).

**TABLE 3:** Association between eating behaviors and obesity based on weight indices.

Eating behaviors	Overweight/Obesity^a^		Abdominal obesity^b^		Central obesity^c^	
	Univariate	Multivariate	Univariate	Multivariate	Univariate	Multivariate
	OR (95% CI)	OR (95% CI)	OR (95% CI)	OR (95% CI)	OR (95% CI)	OR (95% CI)
Picky eating	**0.310** (**0.191–0.501**)***	**0.238** (**0.135–0.420**)***	**0.394** (**0.270–0.576**)***	**0.332** (**0.220–0.502**)***	**0.510** (**0.375–0.693**)***	**0.398** (**0.278–0.571**)***
Food avoidance	**0.241** (**0.138–0.421**)***	**0.188** (**0.098–0.360**)***	**0.338** (**0.220–0.519**)***	**0.306** (**0.195–0.481**)***	**0.463** (**0.329–0.650**)***	**0.389** (**0.264–0.575**)***
FF	0.754 (0.525–1.083)	**0.590** (**0.387–0.899**)*	0.770 (0.577–1.028)	**0.673** (**0.491–0.922**)*	**0.565** (**0.439–0.727**)***	**0.522** (**0.387–0.703**)***
EU	**0.564** (**0.411–0.772**)***	**0.537** (**0.381–0.756**)**	**0.651** (**0.507–0.836**)**	**0.656** (**0.508–0.847**)**	**0.771** (**0.628–0.945**)*	**0.718** (**0.567–0.909**)**
SR	**0.381** (**0.256–0.565**)***	**0.349** (**0.223–0.545**)***	**0.459** (**0.336–0.627**)***	**0.437** (**0.315–0.606**)***	**0.668** (**0.522–0.855**)**	**0.564** (**0.425–0.750**)***
SE	**0.539** (**0.394–0.738**)***	**0.534** (**0.377–0.758**)***	**0.639** (**0.500–0.817**)***	**0.628** (**0.487–0.810**)***	**0.799** (**0.655–0.973**)*	**0.762** (**0.609;0.952**)*
Food approach	**1.830** (**1.207–2.775**)**	**2.172** (**1.334–3.536**)**	**1.648** (**1.169–2.322**)**	**1.929** (**1.326–2.807**)**	**1.429** (**1.065–2.917**)*	**1.759** (**1.241–2.492**)**
EF	**1.915** (**1.388–2.642**)***	**2.207** (**1.515–3.214**)***	**1.569** (**1.234–1.995**)***	**1.710** (**1.317–2.220**)***	**1.343** (**1.112–1.622**)**	**1.669** (**1.327–2.099**)***
EO	**1.536** (**1.009–2.337**)*	**1.690** (**1.045–2.735**)*	**1.506** (**1.058–2.145**)*	**1.651** (**1.135;2.401**)**	1.170 (0.856–1.600)	1.284 (0.898–1.838)
FR	**1.694** (**1.259–2.280**)**	**2.049** (**1.431–2.934**)***	**1.676** (**1.301–2.160**)***	**1.914** (**1.452–2.523**)***	**1.466** (**1.166–1.843**)**	**1.672** (**1.284–2.178**)***
DD	0.915 (0.729–1.148)	0.902 (0.704–1.157)	0.913 (0.761–1.096)	0.930 (0.769–1.123)	0.976 (0.840–1.134)	0.963 (0.810–1.145)

*Note:* The definitions of eating behaviors are explained in discussion section. All *p*‐values were obtained from logistic regression models (*p*‐values < 0.05 are in bold type). Multivariate model was adjusted for variables with *p*‐value < 0.25 in multivariate analysis according to Table S1; age, birth weight (LBW, NBW, and HBW), breastfeeding duration, Kindergarten attendance (< 6 weeks, ≥ 6 weeks), family income, Mother's BMI (underweight, normal weight, overweight, obese), and HEI.

Abbreviations: BAZ, BMI for age‐*z* score; DD, desire to drink; EF, enjoyment of food; EO, emotional overeating; EU, emotional undereating; FF, food fussiness; FR, food responsiveness; HEI, healthy eating index; PE, picky eating; SE, slowness in eating; SR, satiety responsiveness; WC, waist circumference; WHtR, waist circumference to height ratio.

^a^ Overweight/Obesity was categorized based on BMI for age‐*z* score (BAZ) and data are presented as OR (95% CI) compared with normal weight and wasted participants.

^b^ Abdominal obesity was categorized based on WC and data are presented as OR (95% CI) compared with participants without abdominal obesity.

^c^ Central obesity was categorized based on WHtR and data are presented as OR (95% CI) compared with participants without central obesity.

* *p* < 0.05.

** *p* < 0.01.

*** *p* < 0.001.

Food approach, enjoyment of food, and food responsiveness exhibited positive associations with all three obesity indicators. The food approach was linked to 2.1%, 1.9%, and 1.7‐fold higher odds of OW/OB, abdominal, and central obesity, in order (OW/OB: OR = 2.172; 95% CI 1.334–3.536, abdominal obesity: OR = 1.929; 95% CI 1.326–2.807, central obesity: OR = 1.759; 95% CI 1.241–2.492). Emotional overeating was also positively associated with overweight and obesity as well as abdominal obesity, but not central obesity (OW/OB OW/OB: OR = 1.690; 95% CI 1.045–2.735, abdominal obesity: OR = 1.651; 95% CI 1.135–2.401, central obesity: OR = 1.284; 95% CI 0.898–1.838). Desire to drink showed no association with any of the obesity indicators (Table 3).

## 4 Discussion

OW/OB, abdominal, and central obesity were negatively associated with picky eating, food avoidance, and its subsets. But they showed positive associations with food approach, enjoyment of food, and food responsiveness.

Evidence has found some factors as predictors of picky eating: (1) sensory sensitivity, (2) emotional sensitivity, and (3) parental sensitivity. Mealtime sensory experiences—such as oral, olfactory, visual, and even tactile sensations—play a fundamental role in the eating behavior of picky eaters. Sensory sensitivity, characterized by hypersensitivity to taste, texture, smell, and appearance of foods, independently contributes to the rejection of certain food groups, whether new or not, and developing picky eating in preschool and school ages (Kutbi et al. 2019; Steinsbekk et al. 2017). In between, oral texture has been identified as a strong predictor of picky eating across various populations, including youth, healthy children, and children with conditions such as Autism Spectrum Disorder (ASD), anxiety, and Obsessive‐Compulsive Disorder (OCD) (Zickgraf et al. 2022). Additionally, the negative affectivity of children with emotional sensitivity, characterized by emotional undereating, represented another etiology of picky eating (Sandvik et al. 2018). In a longitudinal community study by Hafstad et al. (2013), emotional sensitivity at 1.5 years significantly predicted picky eating at ages 2.5 and 4.5. Also, the negative responses of picky eaters to food—such as higher emotional undereating and lower enjoyment of food—reported by Sandvik et al. (2018), support our finding that picky eating is linked to lower degrees of enjoyment of food and food responsiveness. Another important predictor is parental sensitivity. Parents with high sensitivity and less structuring tend to accept their children's hesitation or rejection of new foods. They avoid offering them again, thereby potentially reinforcing picky eating. In fact, parental sensitivity confirms and supports both sensory and emotional sensitivity of children, which has led to a negative connection with food (Steinsbekk et al. 2017).

The interplay between feelings and behaviors—positive and negative—surrounding food reveals a strong connection between both food avoidance and food approach behaviors with obesity indicators. Enjoyment of food shows a child's enthusiasm about food intake (Sandvik et al. 2018). Food responsiveness reflects how responsive the child is to external food cues (Santos et al. 2011) child is. In support of them, our findings showed more than double the odds of OW/OB in children with enjoyment of food and food responsiveness behaviors.

Emotional undereating and emotional overeating demonstrate whether children decrease or increase their food consumption while facing unpleasant feelings (Santos et al. 2011). In our study, emotional undereating and emotional overeating showed negative and positive associations with obesity indicators, sequentially. Previous studies have supported the positive association of obesity with emotional overeating, but no association for emotional undereating (Dalrymple et al. 2020; Santos et al. 2011). Of note, emotional overeating was not associated with central obesity (WHtR) while linked to abdominal obesity (WC). According to a meta‐analysis among pediatrics (Lo et al. 2016), the discriminatory power of WHtR and WC for cardio‐metabolic risk was the same. But, for the detection of general and central obesity, WHtR was proved to be a better tool in the CASPIAN‐V cohort study (Ejtahed et al. 2019).

Inconsistent with prior research, we found that satiety responsiveness, the child's ability to moderate food intake after eating, and slowness in eating were negatively associated with obesity indicators (Dalrymple et al. 2020; Santos et al. 2011).

According to the parents' answers to CEBQ, there was no significant difference in desire to drink between children with low and high picky eating. However, dietary intake reports indicated that children with high picky eating consumed more sweetened beverages compared to those with low picky eating. This suggests that children with low picky eating may have better control over their desire for sweet drinks, practically. Neither our study nor previous research (Dalrymple et al. 2020; Santos et al. 2011) found any significant association between desire to drink and obesity, likely because the desire level did not accurately reflect actual beverage consumption.

Food fussiness generally indicates a reluctance to eat new or familiar foods (Santos et al. 2011). Questions in this sub‐scale focus on familiar foods more than unfamiliar ones. In agreement with study Dalrymple et al. (2020), our findings revealed approximately 40% lower odds of the obesity indicators associated with food fussiness. Studies by Santos et al. (Santos et al. 2011) and Brown et al. (2018) did not find any association of obesity indicators with food fussiness. It seems that the relationship between food fussiness and obesity may be influenced by confounders, as it became significant after adjusting for related characteristics of children and their families.

Picky eating provides us with a more comprehensive perspective compared to other eating behaviors discussed. Therefore, its association with obesity indicators holds greater importance. We found that picky eating was correlated with approximately 60% lower odds of all three obesity indicators. Our result was in line with two cross‐sectional studies involving over 2000 Chinese school children each (Qiu and Hou 2020; Sun et al. 2020). A large‐scale study on preadolescents conducted by Viljakainen et al. using data from the Finnish Health in Teens (Fin‐HIT) cohort also found 44% lower odds of OW/OB and 2.2‐fold higher odds of underweight among severe picky eaters compared to non‐picky eaters (Viljakainen et al. 2019). In comparison, the stronger link observed in our study could be attributed to the age of participants, encompassing the peak stage of picky eating. According to the robust association between picky eating and wasting found in Viljakainen et al. (2019) and Dubois et al. (2007) studies, this association could go beyond the reduced odds of obesity consequences to higher odds of wasting and malnutrition.

An Italian study on children from 2 to 6 years (Finistrella et al. 2012) found overweight and obese children presented more severe picky eating than their normal‐weight counterparts. The differences in the prevalence of OW/OB (31.5% in Finistrella et al. study vs. 12.4% in our study), the methods used to quantify picky eating (CFQ in Finistrella et al. study vs. CEBQ in our study), and unmeasured population characteristics (such as dietary intakes or income level in Finistrella et al. study) could be the reasons for the conflicting results. According to cohort study Dubois et al. (2007) within the dataset of the Longitudinal Study of Child Development in Québec (LSCDQ), being a picky eater at all three ages of 2.5, 3.5, and 4.5 years increases the risk of underweight by 2.4, but was not associated with overweight. The study by Rohde et al. (2017) did not find any link between picky eating and obesity in normal‐weight children at risk of obesity after a 15‐month follow‐up. Both the Dubois and Rohde studies primarily involved children from privileged families, whereas half of our population comprised underprivileged families. The difference between rates of picky eating and obesity between the children from privileged and underprivileged families, reported by previous research (Chou et al. 2024; Qiu and Hou 2020) could explain the lack of association in the Dubois and Rohde studies. For example, the prevalence of underweight and overweight in the Dubois study was higher and lower than ours, respectively.

Another important factor to be considered is the nutritional intake. In the current study, children with high picky eating consumed higher amounts of fast foods, snacks, and sugar‐sweetened beverages compared to those with low picky eating. Nevertheless, their intake of starch (Bread, rice, and pasta) was remarkably lower, resulting in lower energy intake among high picky eaters. Previous studies have reported similar findings regarding fast foods [11], snacks (Sun et al. 2020; Viljakainen et al. 2019), and vegetables consumption (Viljakainen et al. 2019). A systematic review by Taylor et al. indicated restricted dietary diversity among picky eaters, particularly due to low fruits and vegetables intake (Taylor et al. 2015). In contrast, Rohde et al. study (Rohde et al. 2017) reported similar intakes of energy and food groups between picky eaters and non‐picky eaters, which may result in no relationship between picky eating and obesity. In study Brown et al. (2018) picky eating and obesity weren't associated, but the negative association of picky eating with the diet quality (based on HEI) and the vegetable intake was consistent with our findings. This association between picky eating and dietary habits could extend beyond childhood; a study by Pesch et al. (Pesch et al. 2020) indicated that young adults who were picky eaters in childhood consumed more fast foods, snacks, and sugar‐sweetened beverages but less fruit, vegetables, and whole grains.

The higher severity of picky eating among children who have consumed the highest amounts of UPF was similar to the results of study Vedovato et al. (2021), which indicated that UPF consumption at age four was positively associated with food fussiness by age seven. According to a population‐based cohort (Jansen et al. 2020), parents who have used foods like sweets as rewards at age four contributed to higher levels of picky eating in their children by age nine. Consequently, these parents often prefer to offer ready‐to‐eat industrial foods that children find irresistible. Such parental strategies can make children more hesitant to try new or healthier foods, perpetuating a vicious cycle of limited dietary diversity and intensified picky eating (Vedovato et al. 2021). Focusing on the somatosensory experiences of children, the added fat and refined carbohydrates in UPFs activate the reward‐related neural circuitry, leading to food craving and snacking. This internal process further discourages children from eating staple healthy meals (Schulte et al. 2015). In our study, this discouragement contributed to lower energy intake, despite the high energy density of the UPFs consumed, and ultimately lower odds of obesity.

Literature research showed physical activity has been increasingly replaced by digital media, such as TV and mobile. Yalcin et al. found a direct association between picky eating and more than 2‐h screen time among schoolchildren in Turkey (Yalcin et al. 2022). In our study, picky eating was not associated with preschoolers' screen time, but it was related to having meals in front of a digital screen. Most of the parents reported that their children would only eat meals if placed in front of a digital screen, which varied from TV, smartphone, tablet, or PC, whether for playing or watching. As discussed about sensory sensitivity as a profound pathology of picky eating, screens may distract picky eaters from the overwhelming sensations of food texture or smell. Thus, even if their screen time is similar to that of low picky eaters, most of which coincides with mealtime, as a coping mechanism against sensory hypersensitivity.

## 5 Limitations and Strengths

To the best of our knowledge, this study is the first to evaluate the relationship between obesity and picky eating using a combined score calculated from five related sub‐behaviors. It also included children from all municipalities and educational districts of Shiraz, representing a wide diversity in family incomes and characteristics.

Our research also has some limitations. The cross‐sectional design of the study prevented us from making a causal inference. The assessment of children's eating behaviors relied on parents' reports, which may be influenced by their attitudes and perceptions regarding appetite, food consumption, or even the child's weight status. We did not account for parental feeding practices, which share a causal connection and require further empirical investigation. Another limitation is recalling bias in dietary intake assessment based on FFQ. Although some novel confounders were adjusted in our multivariate analysis, there may still be others remaining unmeasured. Given that the relationship between picky eating and obesity may vary with age and be influenced by cultural differences, future studies should include samples across a broader age range, as well as diverse cultural backgrounds, ethnic origins, and eating styles. This approach will provide a more comprehensive understanding of how these factors affect the association between picky eating and obesity.

## 6 Conclusion

All three obesity indicators were negatively associated with picky eating and food avoidance behaviors but positively associated with food approach behaviors. Picky eaters had lower diet quality and energy intake. Notably, UPF consumption was linked to intensified picky eating. The optimal growth and development of children requires a comprehensive assessment of environmental and psychosocial factors leading to picky eating or other eating difficulties to unveil the vast majority of their constructs. In this context, parental awareness of their feeding practices and beliefs about hunger regulation, along with being attentive to children's emotional and sensory challenges at mealtime, would be a constructive beginning toward nurturing a healthy relationship with nutritious foods.

## Author Contributions

**Melika Mahmoudizadeh:** data curation (equal), formal analysis (equal), investigation (equal), methodology (equal), writing – original draft (equal), writing – review and editing (equal). **Mina Babashahi:** investigation (equal), methodology (equal), writing – review and editing (equal). **Milad Rajabzadeh‐Dehkordi:** methodology (equal), writing – original draft (equal), writing – review and editing (equal). **Mehran Nouri:** data curation (equal), formal analysis (equal), investigation (equal), methodology (equal), writing – original draft (equal), writing – review and editing (equal). **Shiva Faghih:** investigation (equal), methodology (equal), writing – original draft (equal), writing – review and editing (equal).

## Conflicts of Interest

The authors declare no conflicts of interest.

## Supporting information


**Table S1:** The association between baseline variables and odds of overweight and obesity in children.
**Table S2:** The median scores of eating behaviors among study's population based on low and high picky eating.

## Data Availability

The data that support the findings of this study are available from the corresponding author upon reasonable request.
