# Supplementary material for: The Interplay Between Picky Eating, Other Eating Behaviors, and Obesity Indicators in Preschool Children
Source: Food Sci Nutr. 2025 Sep 24;13(9):e70967. doi: 10.1002/fsn3.70967 (PMC12457951; doi:10.1002/fsn3.70967)
Supplement: Supplementary file 1 — Table S1: The association between baseline variables and odds of overweight and obesity in children. Table S2: The median scores of eating behaviors among study's population based on low and high picky eating. [file FSN3-13-e70967-s001.docx]

**Table S1. Association between baseline variables and odds of overweight and obesity in children.**

| **Variables** | **OR** | **CI 95 %** | **P-value** |
| --- | --- | --- | --- |
| **Age** (year) | **1.857** | **1.411-2.446** | **˂0.001** |
| **Physical activity** (score) | 0.806 | 0.512-1.267 | 0.349 |
| **Screen time** (minute) | 0.999 | 0.998-1.001 | 0.603 |
| **Breastfeeding duration** (month) | **0.957** | **0.930-0.984** | **0.002** |
| **Energy** (kcal/day) | 1.000 | 1.000-1.000 | 0.872 |
| **HEI** (score) | 0.973 | 0.936-1.013 | **0.182** |
| **Sex:**  Boy  Girl | Ref.  1.078 | Ref.  0.609-1.909 | 0.797 |
| **Eating in front of a digital screen**:  No  Yes | Ref.  0.918 | Ref.  0.483-1.744 | 0.794 |
| **Kindergarten attendance**:  ˂6 weeks  ≥6 weeks | Ref.  **3.691** | Ref.  **1.917-7.110** | **˂0.001** |
| **Birth weight:** |  |  |  |
| NBW (2500 – 4000 g) | Ref. | Ref. |  |
| LBW (˂2500 g) | 0.781 | 0.266-2.292 | 0.653 |
| HBW (˃4000 g) | 3.711 | 1.214-11.348 | **0.021** |
| **Family income** (rial): |  |  |  |
| ˂100 million | Ref. | Ref. |  |
| 100-200 million | 1.347 | 0.704-2.579 | 0.369 |
| 200-300 million | 1.796 | 0.563-5.729 | 0.323 |
| ≤300 million | 3.689 | 1.512-9.005 | **0.004** |
| **Mother’s age** (year) | 1.016 | 0.962-1.074 | 0.563 |
| **Mother’s BMI** (kg/m^2^): |  |  |  |
| Normal weight | Ref. | Ref. |  |
| Underweight | 0.0 | 0-0 | 0.999 |
| Overweight | 1.013 | 0.511-2.006 | 0.971 |
| Obese | 1.778 | 0.875-3.612 | **0.111** |
| **Mother’s education:** |  |  |  |
| Under diploma | Ref. | Ref. |  |
| Diploma | 0.821 | 0.318-2.124 | 0.685 |
| Upper diploma | 0.989 | 0.410-2.385 | 0.980 |
| **Mother’s job**:  No  Yes | Ref.  1.174 | Ref.  0.601-2.296 | 0.638 |

Abbreviation- OR: odds ratio; CI: confidence interval, HEI: healthy eating index, BMI: body mass index, kg: kilogram, m: meter, kcal: kilocalorie, g: gram.

Obtained from logistic regression.

p-value of more than 0.25 was shown in bold.

**Table S2. Eating behaviors of study’s population based on low and high picky eating.**

| **P value** | **Picky Eating** | | **Total** (n=436) (1.32-4.93) | **Eating behaviors** |
| --- | --- | --- | --- | --- |
|  | **High** (n=219)  (˃3.17) | **Low** (n=217)  (≤3.17) |  |  |
| **˂0.001** | 3.65 (0.53) | 2.69 (0.53) | 3.17 (0.97) | **Picky Eating** ^1^ |
| **˂0.001** | 3.49 ± 0.44 | 2.66 ± 0.41 | 3.08 ± 0.60 | **Food Avoidance**^2^ |
| **˂0.001** | 3.16 (1) | 2.16 (0.67) | 2.66 (1) | FF^1^ |
| **˂0.001** | 3 (1.25) | 2.75 (1.25) | 3 (1.5) | EU^1^ |
| **˂0.001** | 4 (0.8) | 3 (0.8) | 3.4 (1.2) | SR^1^ |
| **˂0.001** | 4 (1.5) | 2.75 (1) | 3.25 (1.5) | SE^1^ |
| **˂0.001** | 2.09 (0.66) | 2.75 (0.85) | 2.41 (0.9) | **Food Approach**^1^ |
| **˂0.001** | 2.75 (1.25) | 4.25 (1) | 3.5 (1.5) | EF^1^ |
| **˂0.001** | 1 (0.75) | 1.5 (1) | 1.25 (0.75) | EO^1^ |
| **˂0.001** | 1.6 (0.6) | 2.4 (1.2) | 2 (1) | FR^1^ |
| 0.851 | 2.66 (2.33) | 2.66 (1.67) | 2.66 (2) | DD^1^ |

Abbreviation-FF: Food Fussiness, SR: Satiety Responsiveness, SE: Slowness in Eating, EU: Emotional Undereating, EF: Enjoyment of Food, EO: Emotional 0vereating, FR: Food Responsiveness, DD: Desire to Drink

^1^Using Mann-Whitney U-test and values are median (IQR).

^2^Using independent sample t-test and values are mean ± SD.
